# MicroRNA-200b-3p promotes endothelial cell apoptosis by targeting HDAC4 in atherosclerosis

**DOI:** 10.1186/s12872-021-01980-0

**Published:** 2021-04-12

**Authors:** Fan Zhang, Naixuan Cheng, Jie Du, Haibo Zhang, Congcong Zhang

**Affiliations:** 1 Beijing Anzhen Hospital, Capital Medical University, Beijing, China; 2 Key Laboratory of Remodeling-Related Cardiovascular Diseases, Ministry of Education, Beijing, China; 3 Beijing Institute of Heart, Lung and Blood Vessel Diseases, Beijing, 100029 China

**Keywords:** Atherosclerosis, Epicardial adipose tissue, MiRNA-200 family, Vascular endothelial cells, MiRNA profile

## Abstract

**Background:**

Epicardial adipose tissue (EAT) shares the same microcirculation with coronary arteries through coronary arteries branches, and contributes to the development of atherosclerosis. MicroRNAs (miRNAs) are involved in the formation of atherosclerosis. However, the alteration of miRNA profile in EAT during atherosclerosis is still uncovered.

**Methods:**

The miRNA expression profiles of EAT from non-coronary atherosclerosis disease (CON, n = 3) and coronary atherosclerosis disease (CAD, n = 5) patients was performed to detect the differentially expressed miRNA. Then the expression levels of miRNA in other CON (n = 5) and CAD (n = 16) samples were confirmed by realtime-PCR. miR-200b-3p mimic was used to overexpress the miRNA in HUVECs. The apoptosis of HUVECs cells was induced by H_2_O_2_ and ox-LDL, and detected by Annexin V/PI Staining, Caspase 3/7 activity and the expression of BCL-2 and BAX.

**Results:**

250 miRNAs were differentially expressed in EAT from CAD patients, which were associated with metabolism, extracellular matrix and inflammation process. Among the top 20 up-regulated miRNAs, the expression levels of miR-200 family members (hsa-miR-200b/c-3p, miR-141-3p and miR-429), which were rich in endothelial cells, were increased in EAT from CAD patients significantly. Upregulation of miR-200 family members was dependent on the oxidative stress. The overexpression of miR-200b-3p could promote endothelial cells apoptosis under oxidative stress by targeting HDAC4 inhibition.

**Conclusions:**

Our study suggests that EAT derived miR-200b-3p promoted oxidative stress induced endothelial cells damage by targeting HDAC4, which may provide a new and promising therapeutic target for AS.

**Supplementary Information:**

The online version contains supplementary material available at 10.1186/s12872-021-01980-0.

## Introduction

Coronary artery disease (CAD) is the most common cardiovascular disease and is characterized by acute coronary syndrome and myocardial infarction. It leads to considerable morbidity and mortality in most countries. The main underlying cause of CAD is coronary atherosclerosis (AS) [1], which is characterized by the initial development of lesions in arterial wall [2]. Sustained exposure to inflammatory microenvironment promotes endothelial cell injury, vascular dysfunction and arterial lumen stenosis, which contribute to the development of an atherosclerotic plaque [3]. However, the pathogenesis of atherosclerosis is still not fully understood.

A recent observation found that coronary AS plaques mainly occur in arterial segments surrounded by epicardial adipose tissue (EAT), leading to the hypothesis that EAT plays a key role in the pathogenesis of CAD [4]. In a human heart, EAT is located in atrioventricular and interventricular grooves, surrounding the major branches of coronary arteries, atria, right ventricular free wall, and left ventricle apex [5]. EAT shares the same microcirculation with the myocardium and coronary arteries. The vascularization of EAT is provided by branches of the coronary arteries, and there was no muscle fascia separating EAT from the myocardium [6]. Since EAT could produce several bioactive molecules, including inflammatory cytokines (IL1-β, IL-6, IL-8, etc*.*) [7], adipokines (FGF-21, leptin, adiponectin, etc*.*) [8], miRNAs that could be paracrinally or vasocrinally secreted to the coronary artery wall [9], EAT is considered to be a large secretosome that regulates physiological and pathophysiological processes in the heart. In fact, the thickness of EAT is an independent risk factor for CAD [10], atrial fibrillation [11], and other cardiovascular disease [12, 13].

MicroRNAs (miRNAs) are 19–22 nucleotides, non-coding and single-stranded ribonucleic acid molecules, and miRNAs are endogenous post transcriptional regulators of gene expression [14–16]. MiRNAs play key roles in the regulation of multiple cellular pathways in myocardial infarction and AS, and are involved in cell proliferation and differentiation, and apoptosis [17, 18]. MiRNAs could be considered as candidate biomarkers and therapeutic target for metabolic disease and CAD [19]. However, the role of miRNAs from EAT in AS has not been fully understood.

In this study, we compared the miRNA expression profiles of EAT from non-CAD and CAD patients, and found that 250 miRNAs (including 53 novel miRNAs) were differentially expressed in EAT from CAD patients, which were associated with metabolic, ECM and inflammation process. Among the top 20 up-regulated miRNAs, we found that miR-200 family members (hsa-miR-200b/c-3p, miR-141-3p and miR-429) were significantly increased in CAD patients. Through bioinformatics analysis and functional verification in vitro, we found that miR-200b-3p could promote the apoptosis of endothelial cells under oxidative stress by targeting HDAC4. Our study may provide a new and promising therapeutic target for AS.

## Methods

### Research population

From July 2017 to March 2019, 29 patients who underwent elective cardiothoracic surgery with informed consent were enrolled at Beijing Anzhen Hospital. 8 underwent cardiac valve replacement surgery or atrial myxoma removal surgery (CTRL group; no history/evidences of CAD/carotid atherosclerosis), and 21 underwent off-pump coronary artery bypass graft surgery (CAD group). The characteristics of the patient are shown in Additional file 1: Table S1. The study was approved by the Ethics Committee of Beijing Anzhen Hospital, and carried out in accordance with The Code of Ethics of the World Medical Association.

### Epicardial adipose tissue collection

As mentioned previously, EATs were collected from all patients during cardiothoracic surgeries (20). EAT samples were obtained near the proximal segment of the right coronary artery, deep to the visceral layer of the pericardium. Tissue samples were immediately frozen on dry ice and then stored at − 80 °C until processing.

### Total RNA extraction

Total RNA was isolated from cell or tissue lysates by using TRIzol reagent (Invitrogen). Briefly, 100 µg tissues was dissolved in 1 ml TRIzol reagent using a gentle MACS dissociator with M tubes and the RNA_02 program (MiltenyiBiotec), then 200 µl/tube chloroform was added and mixed by vortexing, and samples were centrifuged at 12,000 rpm for 15 min at 4 °C. The upper phase enriched with RNA was then mixed with 1.5 volume of isopropanol, and was centrifuged at 12,000 rpm for 15 min at 4 °C. The supernatant was discarded, the precipitate was re-washed with 75% ethanol, followed by another centrifugation of 12,000 rpm for 15 min at 4 °C. The supernatant was discarded, and then the RNA was dissolved in 50ul ddH_2_O. Nanodrop 2000 was used to measure the quality and quantity of RNA.

### MiRNA sequence and bioinformatics analysis

MiRNA sequence and analysis were carried out at BGI company (Shenzhen, China). Small RNA (18-30nt segment) was separated from total RNA of EAT by PAGE gel, and library construction and sequencing were performed. After filtering low-quality tags, clean reads were mapped to the reference genome to identify miRNAs. The miRNA expression level is calculated using Transcripts Per Kilobase Million (TPM). We defined the gene as a DEG (differentially expressed gene) when TPM foldchange ≥ 2 and Q-value ≤ 0.001.TargetScan database was used to predict the target genes of miRNA, and DAVID Bioinformatics Resources 6.8 was used to analyze Gene Ontology (GO) and Kyoto Encyclopedia of Genes and Genomes (KEGG) pathway enrichment of target genes.

### HUVEC culture and miRNA mimic transfection

Human umbilical vein endothelial cells (HUVECs) were purchase from ATCC(ATCC®PCS-100-010). Cultures of HUVECs were grown on fibronectin-coated plates; maintained in EGM (Lonza) supplemented with 10% fetal bovine serum (FBS) and 1% penicillin/streptomycin. micrON^TM^hsa-miR-200b-3p mimic (50 nM) or negative controls (micrON™ miRNA mimic NC #22, RIOBOBIO, Guangzhou, China) were transfected using Lipofectamine 3000 according to manufacturer’s protocol (Invitrogen). Further analysis was performed after 48 h of transfection.

### Realtime-PCR (qRT-PCR)

For miRNAs, the isolated total RNAs were reverse transcribed into complementary cDNAs and qRT-PCR analysis using Bulge-Loop miRNA qRT-PCR kit (Ribobio, Guangzhou, China) with U6 as an internal control. Commercially available primers for Has-miR-200a-3p (miRA0000682), Has-miR-200b-3p (miRA0000318), Has-miR-200c-3p (miRA0000617), Has-miR-141-3p (miRA0000432), Has-miR-429 (miRA1000755) and U6 (miRAN0002-1-100) were bought from the Ribobio (Guangzhou, China). For mRNAs, the isolated total RNAs were reversely transcribed into complementary cDNAs using GoScript™ Reverse Transcriptase Kit (Promega), and then qRT-PCR analysis was performed using SYBR Green PCR Master Mix Reagent Kit (TaKaRa), with GAPDH used as an internal control. The sequences of primers are as follows: Bcl2, 5′-GGTGGGGTCATGTGTGTGG-3′ and 5′-CGGTTCAGGTACTCAGTC ATCC-3′; Bax, 5′-CCCGAGAGGTCTTTTTCCGAG-3′ and 5′-CCAGCCCATGATGG TTCTGAT-3′; HDAC4, 5′-CTTGTGGGTTACCTGGCTCA-3′ and 5′-TCCAACGAGCTCC AAACTCC-3′; GAPDH, 5′-AAGCCTGCCGGTGACTAAC-3′ and 5′-GCGCCCAATACGA CCAAATC-3′. Data were presented as values calculated by 2^−△△Ct^ method.

### Western blot

Proteins from HUVECs were isolated with protein extraction buffer (Thermo Fisher) by incubation on ice for 30 min. After centrifugation at 12,000 rpm for 15 min at 4 °C, supernatants were collected. Protein concentrations were determined by a BCA protein assay kit (Thermo Fisher). Western blotting was performed as described previously [20]. In brief, Total 20 μg of protein samples were loaded at SDS-PAGE electrophoresis gel for running. Then the protein was transferred to NC membrane and blocked in 5% milk powder. Membranes were incubated with specific primary antibodies [anti-BCL2 antibody (Rabbit, 1:1000 dilution), anti-BAX antibody (Rabbit, 1:1000 dilution), anti-HDAC4 antibody (Rabbit, 1:1000 dilution), anti-GAPDH antibody (mouse, 1:1000 dilution) and anti-β-Actin antibody (mouse, 1:1000 dilution), from Cell Signaling Technology] at 4 °C overnight. Then the membranes were washed and incubated with a secondary antibody (Li-COR Biosciences, Lincoln, NE) conjugated with IRD800 at room temperature for 1 h in the dark. The membranes were then washed and analyzed by the Odyssey software system (Li-COR). The ratio of the protein change was normalized to GAPDH.

### Proliferation assay

To detect the proliferation of cultured HUVECs after stimulation, EdU (1 μM) was added to the culture medium 4 h before the end point of 24 h stimulation. EdU staining was then performed according to the manufacturer’s instructions of EdU Apollo488 In Vitro Kit (Ribobio) and analyzed by High-Content Imaging System (ImageXpress Micro, Molecular Device, USA).

### Apoptosis assay

In order to detect apoptosis of cultured HUVEC after stimulation, Annexin V and PI staining were performed according to the manufacturer’s instructions of FITC Annexin V Apoptosis Detection Kit (BD Bioscience) and analyzed by flow cytometry (BD Fortessa). The activity of Caspase 3 and Caspase 7 was detected by the Caspase-Glo® 3/7 Assay System (Promega) according to the manufacturer’s instructions.

### Statistical analysis

Data processing was performed using SPSS21.0 software. The numeration data was expressed as a percentage, and the χ^2^ test was used for comparison between groups; the measurement data were expressed as mean ± standard deviation, and the unpaired *t* test was used for comparison between groups. *p* < 0.05 was considered to have a statistically significant different between groups.

## Results

1. Distinct miRNA expression profiles in CTRL and CAD EAT samples

**Fig. 1:**
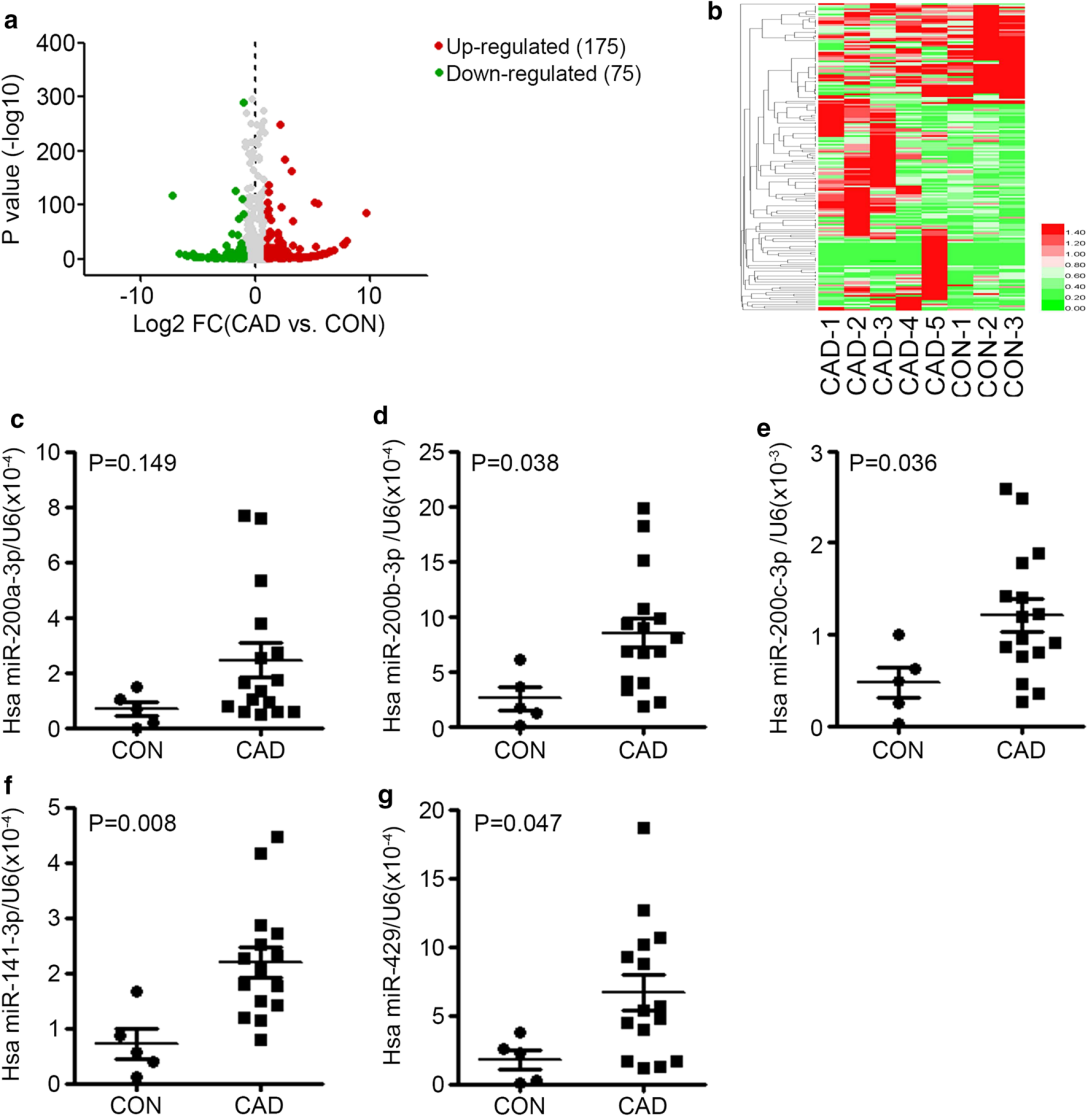
The up-regulation of miR-200 family members in EAT from CAD patients. **a** The Volcano plot of miRNA expression fold change between CAD and CON (x axis, log2 fold change; y axis, *p* value significance). Up-regulated genes (Log2 FC > 1) in CAD samples are indicated by a red dot, and Down-regulated genes (Log2 FC < − 1) in CAD samples are indicated by a green dot. **b** Heat-map of the 250 differentially expressed transcripts. Red color indicated higher expression level; green color indicated lower expression level. **c**–**g** The expression levels of hsa-miR-200a-3p (**c**), hsa-miR-200b-3p (**D**), hsa-miR-200c-3p (**e**), hsa-miR-141-3p (**f**) and hsa-miR-429 (**g**) in EAT from CAD patients (n = 16) and CON patients (n = 5) were accessed by qRT-PCR, and normalized with U6. Unpaired *t* test was used for comparison between groups

In order to perform an unbiased survey of changes in the miRNA transcriptome of EAT from CAD patients, 5 CAD samples and 3 CTRL samples were randomly selected from the established sample pool, and then used for miRNA sequencing. As shown in Additional file 1: Table S2, an average of 1300 known miRNAs and 49 novel miRNAs were detected in each sample, and the average mapping rate (mapped to sRNA database such as miRBase, Rfam) of each sample was 97.19%, indicating that the equal level of sequencing quality of each sample. Compared to CTRL samples, there were a total of 250 differentially expressed miRNAs (FC > 2, *p* < 0.001) in CAD samples. Despite the 53 novel miRNAs, among these 197 known miRNAs, 120 were up-regulated in CAD samples and 57 were down-regulated (Fig. 1a). Unsupervised hierarchic clustering of the two groups was performed on the 250 differently expressed miRNAs and shown as heatmap (Fig. 1b).

To better understand the role of EAT in CAD, TargetScan were used to predict targets for differentially expressed miRNAs in CAD samples. Then GO enrichment analysis was used to reveal the function of target genes. Potential affected GO pathways (Corrected *p* value < 0.05) include: retinoid binding, isoprenoid binding, cytokine activity, extracellular matrix (ECM) structural constituent, G-protein coupled receptor binding, chemokine activity, complement component C1q binding, cytokine receptor binding and antioxidant activity (Additional file 1: Table S3).

These results indicated that miRNA expression profiles in EAT of CAD patients have changed, and these miRNAs were associated with ECM, inflammation and oxidant stress.

2. Upregulation of the miRNA-200 family in EAT of CAD patients

To reveal a new functional miRNA in AS, the top 20 up-regulated and down-regulated miRNAs in CAD patients were listed in Additional file 1: Table S4 and Table S5 separately. We found that almost all members of miR-200 family were among the top 20 up-regulated miRNAs (Additional file 1: Table S4). For the lower expression levels of hsa-miR-200b-5p and hsa-miR-200a-5p, we further verified the differential changes of hsa-miR-200b-3p, hsa-miR-429, hsa-miR-141-3p, hsa-miR-200a-3p, hsa-miR-200c-3p by qRT-qPCR in other 5 CTRL samples and 16 CAD samples. The data showed the expression of hsa-miR-200b-3p (*p* = 0.038), hsa-miR-200c-3p (*p* = 0.036), hsa-miR-141-3p (*p* = 0.008) and hsa-miR-429 (*p* = 0.047) were up-regulated in CAD samples, while hsa-miR-200a-3p was not significantly up-regulated (*p* = 0.149) (Fig. 1c–g). These data demonstrated that the miR-200 family members from EAT may be involved in the pathology of AS.

3. Oxidative stress response promotes the expression of miR-200 family in endothelial cells

**Fig. 2:**
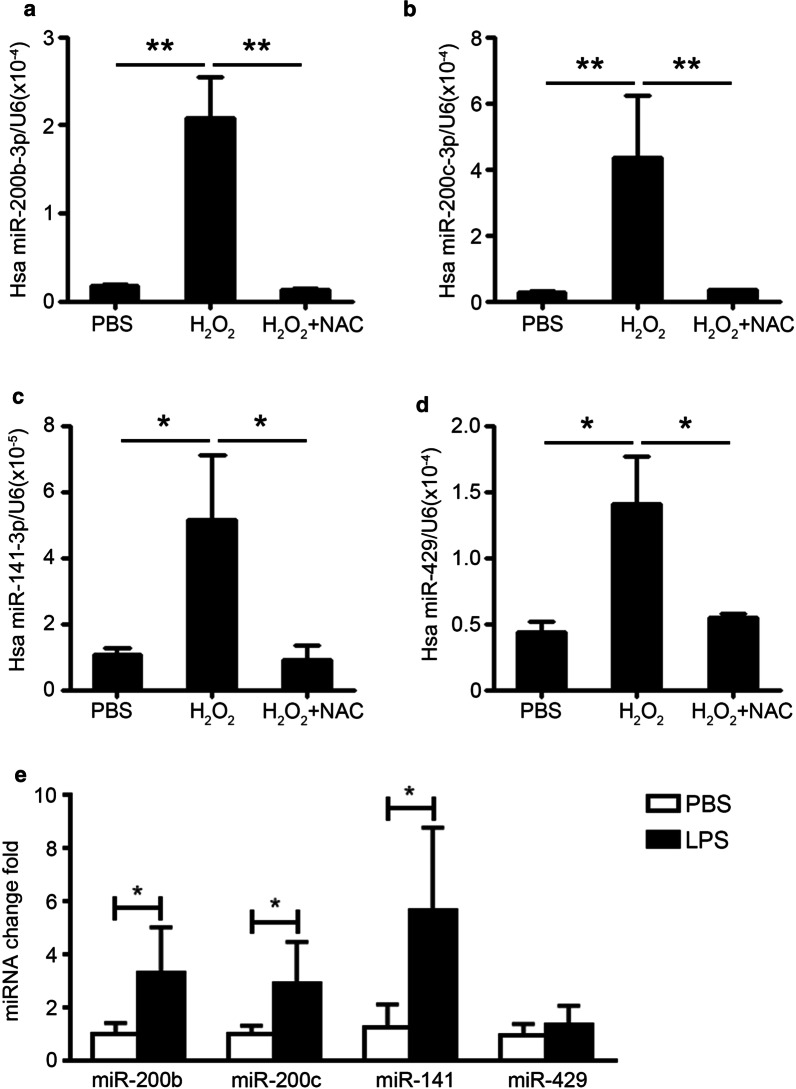
Oxidative stress response promotes the expression of miR-200 family in endothelial cells. **a**–**d** The expression levels of hsa-miR-200b-3p (**a**), hsa-miR-200c-3p (**b**), hsa-miR-141-3p (**c**) and hsa-miR-429 (**d**) in HUVECs with H_2_O_2_ (100 μM) and NAC (10 μM) were accessed by qRT-PCR, and normalized with U6. **e** HUVECs were stimulated with LPS (1 μg/ml) for 12 h, the expression levels of hsa-miR-200b-3p, hsa-miR-200c-3p, hsa-miR-141-3p and hsa-miR-429 were accessed by qRT-PCR, and normalized with U6. Unpaired *t* test was used for comparison between groups, *p < 0.05, **p < 0.01

It is reported that the miR-200 family members are mainly expressed in endothelial cells and are associated with the process of proliferation and endothelial-mesenchymal transition [21, 22]. To elucidate the up-regulation mechanism of miR-200 family in endothelial cells, we stimulated the HUVECs by H_2_O_2_, an analogue of ROS produced by oxidative stress. The qRT-PCR data showed that the expression levels of hsa-miR-200b-3p, hsa-miR-200c-3p, hsa-miR-141-3p and hsa-miR-429 were all significantly increased in HUVECs at 6 h after stimulation, while this increase was blocked by ROS scavenger NAC (Fig. 2a–d). We also pretreated the HUVECs with lipopolysaccharide (LPS) (1 μg/ml), an oxidative stress response inducer in AS. We found that LPS could increase the expression of miR-200b-3p (3.30 folds vs PBS), miR-200c-3p (2.90 folds) and miR-141-3p (5.67 folds) in HUVECs significantly, while the expression of miR-429 was increased slightly by LPS (1.36 folds, *p* = 0.30) (Fig. 2e). The results indicated that oxidative stress response in injured endothelial cells could up-regulate the expression of miR-200 family.

4. miR-200b could increase the apoptosis of endothelial cells

**Fig. 3:**
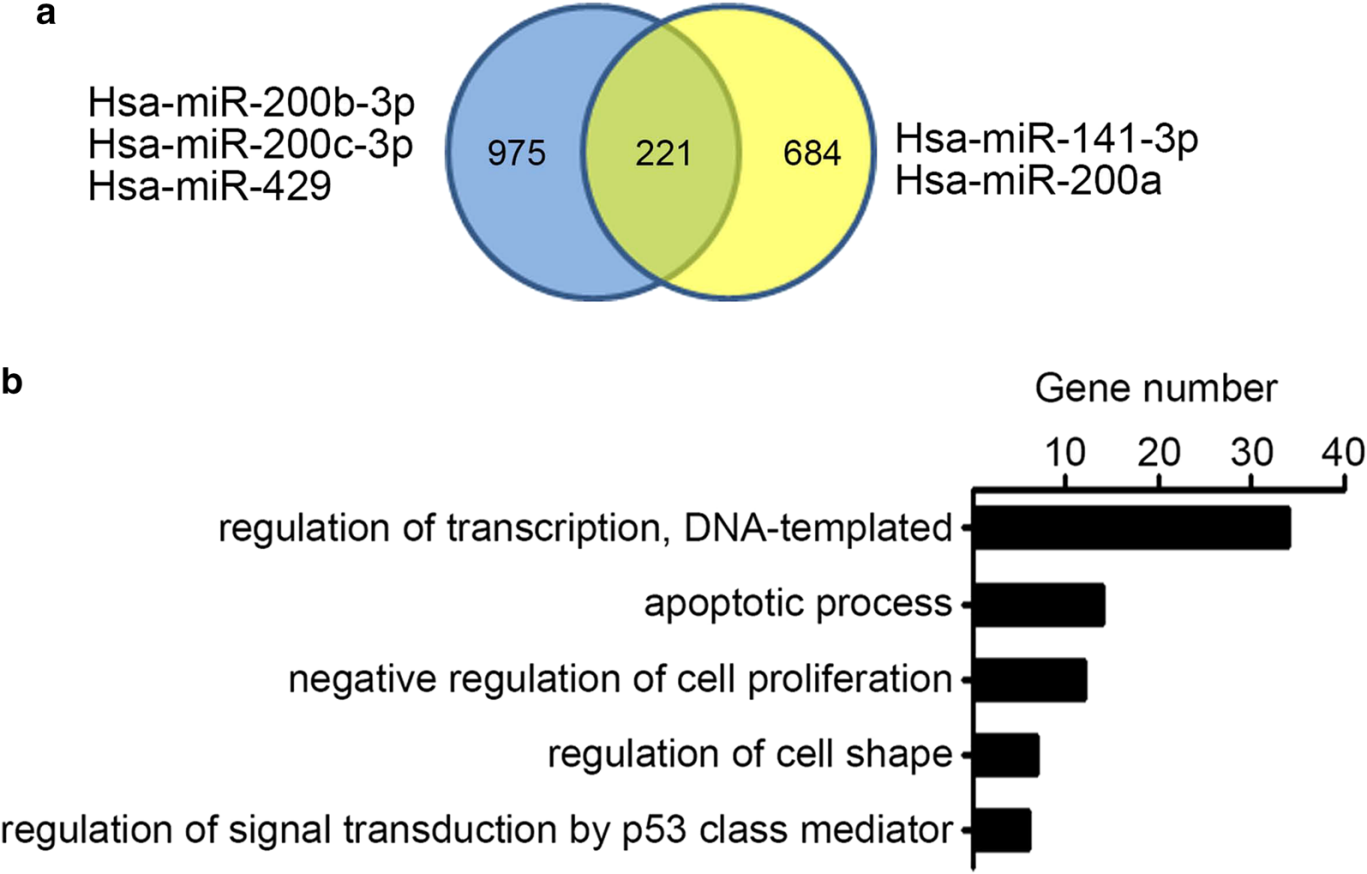
The function prediction of miR-200 family. **a** The targets genes of miR-200 family members, which was predicted by TargetScan database. Then the intersection targets of two sub-group were analyzed. **b** The top 5 enriched KEGG pathway of the intersection targets were analyzed by DAVID database

To clarify the effect of miR-200 family on endothelial cell, we analyzed the predicted target genes of miR-200 family by TargetScan. The miR-200 family members can be divided into two clusters according to the different seed sequences [22]. We found that there were 221 common target genes (Fig. 3a). Then KEGG pathway analysis of these target genes revealed that the enriched pathways included regulation of transcription, apoptosis process, negative regulation of cell proliferation, regulation of cell shape, regulation of signaling transduction by P53 (Fig. 3b).

**Fig. 4:**
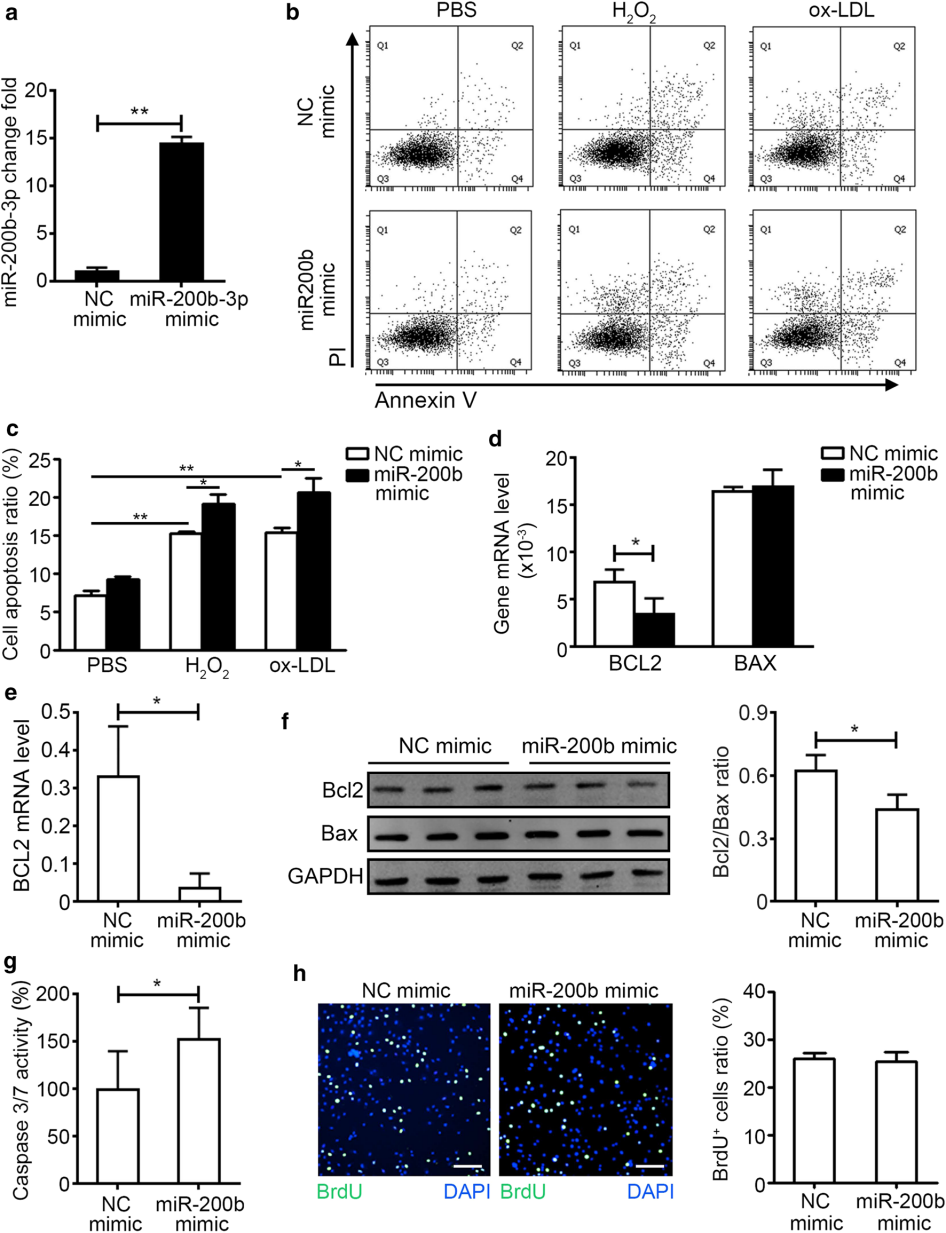
Overexpression of hsa-miR-200b-3p exacerbates cell apoptosis induced by H_2_O_2_ and ox-LDL. **a** HUVECs were transfected with negative control (NC) mimic (50 nM) and hsa-miR-200b-3p (miR-200b) mimic (50 nM) for 24 h, then qRT-PCR for hsa-miR-200b-3p was used to access the transfection efficiency (n = 4 for each group). **b** HUVECs were transfected with NC mimic and miR-200b mimic for 24 h, then cells were stimulated with H_2_O_2_ (50 μM) or ox-LDL (100 μg/ml) for 6 h. The apoptosis of HUVECs was detected by Annexin V and PI staining. **c** The apoptosis ratio of HUVECs was calculated (n = 3 for each group). **d** The mRNA levels of anti-apoptosis gene BCL2 and pro-apoptosis gene BAX in H_2_O_2_ stimulated HUVECs were examined by qRT-PCR (n = 4 for each group). **e** The mRNA levels of BCL2 in ox-LDL stimulated HUVECs were examined by qRT-PCR (n = 4 for each group). **f** The protein levels of BCL2 and BAX in ox-LDL stimulated HUVECs were examined by western blot, and normalized with GAPDH. The right histogram was the ratio of BCL2 and BAX (n = 3 for each group). Full-length blots/gels are presented in Additional files 2 and 3: Figure S1 and S2. **g** The activity of Caspase3/7 in H_2_O_2_ stimulated HUVECs was examined by Caspase-Glo® 3/7 Assay System (n = 6 for each group). **h** HUVECs were transfect with NC mimic and miR-200 mimic for 24 h, then the proliferation rate of HUVECs was accessed by EdU staining (Bar, 50 μm, n = 6 for each group). Unpaired *t* test was used for comparison between groups, *p < 0.05, **p < 0.01

miR-200b-3p was the most up-regulated member in miR-200 family (Additional file 1: Table S4), so we examined the effect of miR-200b-3p overexpression by mimic on the proliferation and apoptosis of HUVECs for further study. Firstly, the overexpression of miR-200b-3p by mimic was confirmed by qRT-PCR (Fig. 4a). Secondly, the NC mimic or miR-200b-3p mimic transfected HUVECs were stimulated by H_2_O_2_ or oxidized low density lipoprotein (ox-LDL) for 6 h, we found that the apoptosis ratio of HUVECs was increased by miR-200b-3p mimic transfection, which was accessed by Annexin V and PI staining (Fig. 4b, c). At the same time, we also found that the expression level of anti-apoptosis gene Bcl2 was decreased and the expression level of pro-apoptosis gene Bax was not changed in miR-200-3p overexpression HUVECs under H_2_O_2_ stimulation (Fig. 4d). The mRNA and protein levels of Bcl2 were also decreased in miR-200-3p overexpression HUVECs under ox-LDL stimulation (Fig. 4e, f, Additional files 2 and 3: Figure S1, S2). The activity of Caspase 3/7 was also increased in miR-200-3p overexpression HUVECs under H_2_O_2_ stimulation (Fig. 4g). The proliferation ratio of HUVECs was not directly affected by miR-200b-3p mimic transfection (Fig. 4h). These data indicated that miR-200b could increase endothelial cell apoptosis under ox-LDL stimulation.

5. HDAC4 is the target gene of miRNA-200b-3p

**Fig. 5:**
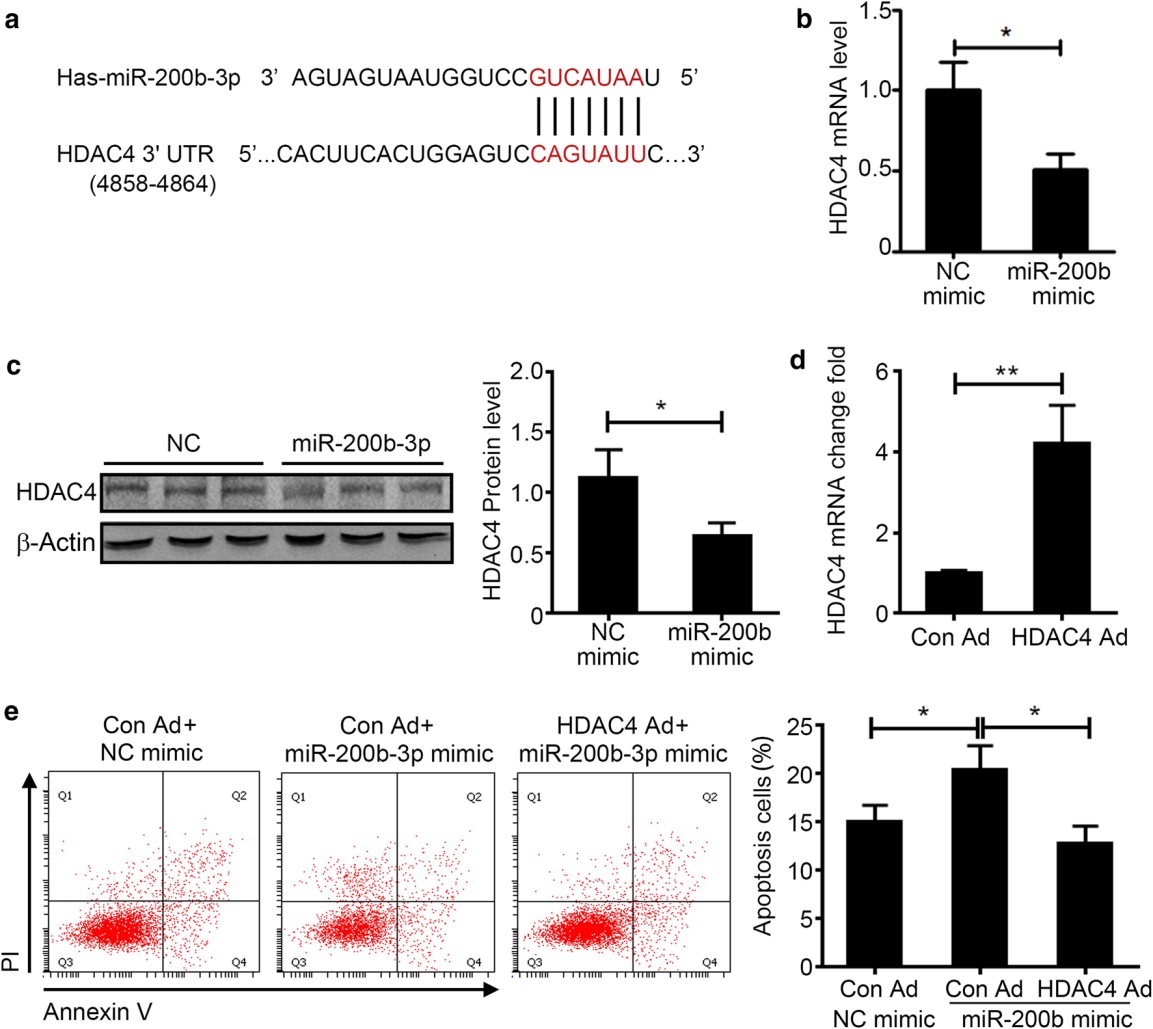
HDAC4 is the target gene of miRNA-200b-3p. **a** Identification the binding sites of hsa-miR-200b-3p in the HDAC4 3′-UTR (Red). **b** HUVECs were transfect with NC mimic and miR-200b mimic for 24 h, the mRNA level of HDAC4 in HUVECs were examined by qRT-PCR, and normalized with GAPDH. (n = 4 for each group). **c** The protein level HDAC4 in HUVECs was examined by western blot, and normalized with β-Actin (n = 3 for each group). Full-length blots/gels are presented in Additional file 4: Figure S3. **d** HUVECs were transfect with NC/miR-200b mimic with control adenovirus (Con-Ad) or HDAC4 over-expression adenovirus (HDAC4-Ad) for 48 h, the expression of HDAC4 was accessed by qRT-PCR (n = 4 for each group). **e** Con-Ad or HDAC4-Ad transfected HUVECs were stimulated with ox-LDL for 6 h. The apoptosis rate of HUVECs was accessed by Annexin V and PI staining (n = 4 for each group). Unpaired *t* test was used for comparison between groups, *p < 0.05

HDAC4, one of the predict target genes, is related to cell apoptosis and has a binding site in the mRNA 3′UTR that is complementary to the miR-200b-3p seed, and the predicted binding site is highly conserved among vertebrates (Fig. 5a). The mRNA level of HDAC4 was decreased in miR-200b-3p mimic transfected HUVECs significantly, which was accessed by qRT-PCR (Fig. 5b). At the same time, the protein level of HDAC4 in miR-200b-3p mimic transfected HUVECs was also significantly reduced, which was accessed by Western Blot (Fig. 5c, Additional file 4: Figure S3). We then transfected the HDAC4 overexpression adenovirus (HDAC4-Ad) and control adenovirus (Con-Ad) to the HUVECs, and the expression of HDAC4 was increased in HDAC4-Ad transfected HUVECs, which confirmed the transfection efficiency (Fig. 5d). Then we found that HDAC4 overexpression could reduce the increased apoptosis induced by miR-200b-3p mimic transfection (Fig. 5e). Therefore, we concluded that HDAC4 was the direct target gene of miRNA-200b-3p.

## Discussion

**Fig. 6:**
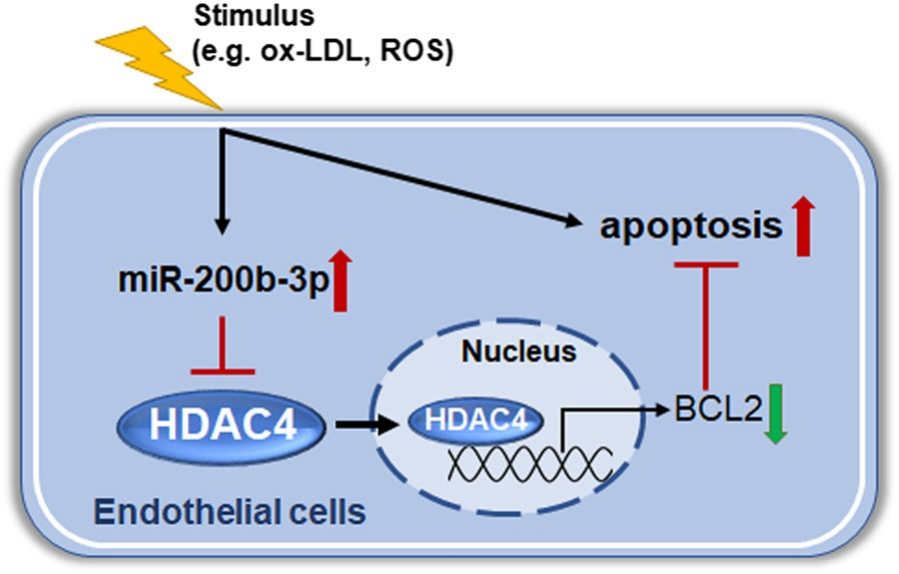
The illustration of the explained mechanisms for miR-200b-3p in endothelial apoptosis

MiRNAs are the key regulators in atherosclerosis and other cardiovascular diseases. In this study, we revealed that the miRNA profile in EAT from CAD patients has changed. Among the differentially expressed miRNAs, the up-regulation of four miRNA-200 family members was confirmed in other CAD samples. The up-regulation of miRNA-200b-3p was induced by oxidative stress response, and could promote the apoptosis of endothelia cells under ox-LDL stimulation by targeting HDAC4 (Fig. 6).

EAT is an atypical fat depot around the heart and plays a putative role in the development of AS [23]. EAT from CAD patients has been reported to be characterized by enhanced inflammation and a suppression of anti-inflammatory miRNAs [24, 25]. miR-103-3p was down-regulated in EAT from CAD patients, and was associated with negative regulation of inflammatory pathways and promotion of adipocyte metabolism by targeting CCL13 inhibition [25]. In our miRNA-sequencing results, the expression of hsa-miR-103a-3p was also decreased in EAT from CAD patients. In addition, we also found that pro-inflammatory miRNAs (miR-146a-5p [26], miR-155-3p [26], miR-206 [27] and miR-223-5p [28]) were up-regulated and anti-inflammatory miRNA Let-7i [29] and miR-127-5p [30] were down-regulated. These data suggested that AS was an inflammatory process and the EAT pro-inflammatory environment could promote the progress of AS in the coronary artery.

EAT is rich in blood vessels and shares the same circulation environment with the coronary arteries. Under the pro-atherosclerosis environment, the changes profile of endothelial cell in EAT could reflect the changes of coronary endothelial cells to a certain extent. At the same time, endothelial cells of EAT could also release miRNAs encapsulated exosomes to the circulation, and EAT derived miRNAs could affect the functions of other cells after being taken up [31–33]. The apoptosis of endothelial cells was considered as the basis of AS pathophysiology. It has been reported that miR-34a [34], miR-127 [35], miR-429 [36], and miR-122-5p [37] can promote EC apoptosis in vitro or in vivo. In this study we also found increased expression of miR-429, and miR-122-5p in EAT of CAD patients, which could reflect the increased endothelial cells apoptosis in AS. The miR-200 family members include miR-200a, miR-200b, miR-200c, miR-141 and miR-429. Among the top 20 up-regulated miRNA, we found that almost all the miR-200 family members were increased in EAT of CAD patients. miR-200b-3p and other members of miR-200 family are highly expressed in endothelial cells. There were no reports about the serum levels of miR-200 family in CAD patient. However, it was reported that the plasma levels of miR-200b were significantly elevated in stroke patients with vulnerable carotid atherosclerosis plaque [38]. Another study also showed that the miR-200c was also up-regulated in carotid plaques and is higher in unstable plaques than stable plaques [39]. These evidences indicated that the miR-200 family is involved in the formation of atherosclerosis.

It was reported that the members of miR-200 family could participate in cell proliferation, differentiation and apoptosis [40]. The functions of common target genes of all the miR-200 family members were also enriched in apoptosis process and negative regulation of cell proliferation pathways. The expression levels of miR-200 family members were significantly increased in the mouse models of hypoxia-reoxygenation [41]. Intracellular oxidative stress response caused by hypoxia-reoxygenation is essential for promoting the increased expression of miR-200 family. In the process of AS, endothelial cell apoptosis can be induced by a variety of atherogenic factors, such as angiotensin II, hyperglycemia, ox-LDL and nicotine intake [42–44]. These factors could induce the oxidative stress response in the endothelial cells. In this study, the expression levels of miR-200 family members were increased after H_2_O_2_ and LPS stimulation and ROS scavenger NAC prevented this increase, further proved that the up-regulation mechanism of miR-200 family during AS was oxidative stress. Besides endothelial cells, the miR-200 family also played a critical role in vascular smooth muscle cells (VSMC). Type 2 diabetes is a risk factor of CAD, it was reported that miR-200 family members miR-200b, miR-200c, and miR-429 were upregulated in VSMC and aortas from type 2 diabetic db/db mice, and miR-200b, miR-429 mimics and target gene Zeb1 siRNAs increased the expression of inflammatory genes COX-2 and MCP-1 as well as monocyte binding to VSMC [45]. Smoking is another risk factor of CAD, it was also reported that nicotine treatment could induce the up-regulation of miR-200b in VSMC, which in turn facilitated VSMC dysfunction through targeting RhoGDIA directly [46].

In this study, we found that overexpression of miR-200b-3p in HUVEC cells could increase the apoptosis ratio of cells under H_2_O_2_ or ox-LDL stimulation by targeting HDAC4 inhibition. HDAC4, a member of the class IIa histone deacetylases (HDACs), is an important regulator of gene expression as part of transcriptional corepressor complexes. It was reported that HDAC4 participated in the apoptosis process of various cells. Overexpression of HDAC4 or increased nuclear location in ovarian cancer cell line-SKOV3 could promote the mRNA expression of BCL2 and inhibited the cell apoptosis [47]. Besides the target gene BCL2, HDAC4 could also inhibit the apoptosis by other pathway. Recent studies have shown that the HDAC4 could bind to activating transcription factor 4 (ATF4), a key transcriptional factor of ER stress that inhibited the expression of CHOP and TRB3 and apoptosis in HEK293T cells [48]. In Hela cells, HDAC4 RNAi could induce mitotic arrest followed by caspase-dependent apoptosis [49]. Through H_2_O_2_ treatment, HDAC4 translocated from the cytoplasm to the nucleus and inhibited the transcription of peroxisome proliferator-activated receptor γ (PPARγ) which was an essential factor for neuron survival [50]. In this study, we showed that over expression of miR-200b-3p in HUVEC cells could reduce the expression of HDAC4, while adenovirus-mediated HDAC4 expression could rescue the increased apoptosis of miR-200b-3p overexpressed HUVEC cells.

## Limitations

This study has several potential limitations. First, the sample size for realtime-PCR verification of RNA-seq data is relatively small. A larger population study with large sample size may improve the reliability of the data in future. Second, only the mRNA and protein levels of HDAC4 were examined after miR-200b-3p overexpression, the dual luciferase reporter assays for the 3′ UTR binding site of HDAC4 with miR-200b-3p are needed to perform in future. Third, only the function of miR-200b-3p was verified in this study, while the functions of other miR-200 family members were predicted based on bioinformatics analysis. We found some similar functions of other miR-200 members with miR-200b-3p. Further experimental studies for other members are needed to clarify the potential functions in atherosclerosis.

## Future directions

The functions of miR-200 family in atherosclerosis in vivo should be verified by genetic knockout mice or agomir/antagomir. The correlation of miR-200 family members in serum or EAT with the indexes, such as the degree of coronary atherosclerosis, major adverse cardiovascular events and so on, should also be explored. Our study contributes to explore new potential biomarkers and therapeutic targets for atherosclerosis.

## Conclusion

Our study showed that the miRNA profile in EAT was changed during AS, and up-regulated miR-200b-3p could promote the apoptosis of endothelial cells by targeting HDAC4 inhibition.

## Supplementary Information

**Additional file 1:** Supplementary Tables.

**Additional file 2:** Figure S1. The full-length blot for figure 4F.

**Additional file 3: **Figure S2. The full-length blot for Figure 4F.

**Additional file 4:** Figure S3. The full-length blot for Figure 5C.

## Data Availability

The RNA-sequencing datasets in this study are deposited in GEO database (GSE160197), and other data used and analyzed during the current study are available from the corresponding author on reasonable request.
