# Supplementary material for: MicroRNA-200b-3p promotes endothelial cell apoptosis by targeting HDAC4 in atherosclerosis
Source: BMC Cardiovasc Disord. 2021 Apr 12;21:172. doi: 10.1186/s12872-021-01980-0 (PMC8042726; doi:10.1186/s12872-021-01980-0)
Supplement: Supplementary file 1 — Additional file 1: Supplementary Tables. [file 12872_2021_1980_MOESM1_ESM.docx]

**MicroRNA-200b-3p promotes endothelial cell apoptosis by targeting HDAC4 in atherosclerosis**

Fan Zhang^1^, Naixuan Cheng^1^, Jie Du^1,2,3^, Haibo Zhang^1^*, Congcong Zhang^1,2,3^*

^1^Beijing Anzhen Hospital, Capital Medical University;

^2^Key Laboratory of Remodeling-related Cardiovascular Diseases, Ministry of Education; ^3^Beijing Institute of Heart, Lung and Blood Vessel Diseases, Beijing, 100029, China

*Correspondence to:

Dr. Haibo Zhang, Beijing Anzhen Hospital, Capital Medical University, China. Tel: +86-10-64456868, E-mail: zhanghb2318@163.com;

Dr. Congcong Zhang, Beijing Anzhen Hospital, Capital Medical University, China. Tel: +86-10-64456029, E-mail: zcc_anzhen@163.com

**Table S1. Baseline Characteristics of non-CAD Control Subjects and CAD Patients**

| **Parameter** | **CTRL (n=8)** | **CAD (n=21)** | **P value** |
| --- | --- | --- | --- |
| Age, y | 48±12 | 62±9 | 0.019 |
| Male, n (%) | 4(50) | 17(81) | 0.163 |
| BMI, Kg/m2 | 23.91±4.72 | 26.83±4.47 | 0.033 |
| **Physical Examination** | | | |
| Systolic blood pressure, mm Hg | 119.33±8.06 | 120.94±10.59 | 0.710 |
| Diastolic blood pressure, mm Hg | 71.83±7.08 | 68.56±7.94 | 0.372 |
| Heart rate, bpm | 69.45±8.21 | 76.39±10.57 | 0.079 |
| **Metabolism** | | | |
| TCHO, mmol/L | 3.95±0.66 | 3.71±1.02 | 0.540 |
| LDL, mmol/L | 2.31±0.67 | 2.19±0.93 | 0.728 |
| HDL, mmol/L | 1.18±0.21 | 1.04±0.47 | 0.331 |
| Triglycerides, mmol/L | 1.19±0.37 | 1.53±0.58 | 0.027 |
| Fasting blood glucose, mmol/L | 4.97±0.32 | 6.32±2.01 | 0.019 |
| **Cardiac Function** | | | |
| LVEF, % | 64±6.90 | 63.75±5.76 | 0.939 |
| LVFS,% | 35.17±5.56 | 35.00±4.10 | 0.949 |
| **Anamnesis** | | | |
| Hypertension, n (%) | 1(12.5) | 10(47.6) | 0.100 |
| Angina, n (%) | 0(0) | 15(71.4) | 0.001 |
| Diabetes, n (%) | 0(0) | 4(19.0) | 0.552 |
| Hyperlipidemia, n (%) | 1(12.5) | 5(23.8) | 0.647 |
| **Theraphy** | | | |
| Fibrates, n (%) | 0(0) | 0(0) | >0.999 |
| Statins, n (%) | 0(0) | 15(71.4) | 0.001 |
| Biguanides, n (%) | 0(0) | 2(9.5) | >0.999 |
| Sulfonylureas, n (%) | 1(12.5) | 2(9.5) | >0.999 |
| Thiazolidinediones, n (%) | 0(0) | 2(9.5) | >0.999 |
| Insulin, n (%) | 0(0) | 5(23.8) | 0.283 |
| ACE inhibitors, n (%) | 0(0) | 1(4.8) | >0.999 |
| Sartans, n (%) | 0(0) | 1(4.8) | >0.999 |
| Calcium Channel Antagonists, n (%) | 0(0) | 13(61.9) | 0.003 |
| Beta Blockers, n (%) | 2(25.0) | 17(80.9) | 0.009 |
| Alpha Blockers, n (%) | 0(0) | 0(0) | >0.999 |
| Anticoagulant, n (%) | 1(12.5) | 16(76.2) | 0.003 |
| Diuretic, n (%) | 7(87.5) | 3(14.3) | 0.005 |

TCHO, total cholesterol; HDL, high-density lipoprotein; and LDL, low-density lipoprotein; LVEF, left ventricular ejection fraction; LVFS, left ventricular fractional shortening; ACE, angiotensin-converting enzyme.

**Table S2. Alignment statistics of miRNA count and tags align to reference genome**

| **Sample name** | **Known miRNA count** | **Novel miRNA count** | **Total**  **tag** | **Mapped**  **tag** | **Percentage**  **(%)** |
| --- | --- | --- | --- | --- | --- |
| CAD1 | 1223 | 48 | 26559074 | 25937556 | 97.66 |
| CAD2 | 1239 | 72 | 22420118 | 21769917 | 97.10 |
| CAD3 | 1243 | 75 | 25502934 | 24146357 | 94.68 |
| CAD4 | 1351 | 43 | 25788826 | 25025470 | 97.04 |
| CAD5 | 1304 | 32 | 26301498 | 25781958 | 98.02 |
| CON1 | 1259 | 38 | 26911858 | 26430198 | 98.21 |
| CON2 | 1417 | 51 | 26305657 | 25508519 | 96.97 |
| CON3 | 1360 | 35 | 28528153 | 27915404 | 97.85 |

**Table S3. Top 10 enriched GO pathways**

| **#** | **Gene Ontology term**  **(molecular function)** | **Cluster frequency**  **(total 14949 genes)** | **Corrected P-value** |
| --- | --- | --- | --- |
| 1 | retinoid binding | 48 (0.3%) | 6.85E-11 |
| 2 | isoprenoid binding | 49 (0.3%) | 9.32E-11 |
| 3 | cytokine activity | 186 (1.2%) | 5.59E-07 |
| 4 | extracellular matrix structural constituent | 97 (0.6%) | 0.00031 |
| 5 | G-protein coupled receptor binding | 233 (1.6%) | 0.01187 |
| 6 | chemokine activity | 40 (0.3%) | 0.01206 |
| 7 | complement component C1q binding | 9 (0.1%) | 0.01912 |
| 8 | cytokine receptor binding | 257 (1.7%) | 0.03033 |
| 9 | unfolded protein binding | 84 (0.6%) | 0.03408 |
| 10 | antioxidant activity | 70 (0.5%) | 0.03587 |

**Table S4. Top 20 upregulated miRNA in EAT samples of CAD patients**

| **miRNA id** | **Expression**  **(CON)** | **Expression**  **(CAD)** | **log2Ratio**  **(CAD/CON)** | **P value** | **Q value** |
| --- | --- | --- | --- | --- | --- |
| hsa-miR-205-3p | 0.001 | 4.4548 | 9.652625 | 2.83E-86 | 1.02E-85 |
| hsa-miR-205-5p | 1.066667 | 504.54 | 9.049991 | 0 | 0 |
| hsa-miR-6510-3p | 0.001 | 1.1528 | 7.701535 | 4.03E-29 | 1.01E-28 |
| hsa-miR-383-5p | 0.24 | 20.098 | 6.577935 | 0 | 0 |
| hsa-miR-383-3p | 0.001 | 0.4388 | 6.305606 | 6.01E-13 | 1.07E-12 |
| hsa-miR-200b-3p | 2.363333 | 143.982 | 6.09253 | 0 | 0 |
| hsa-miR-429 | 2.623333 | 106.856 | 5.509423 | 0 | 0 |
| hsa-miR-200b-5p | 0.096667 | 3.966 | 5.47834 | 3.10E-104 | 1.19E-103 |
| hsa-miR-6131 | 0.027333 | 0.9962 | 5.267132 | 9.38E-24 | 2.16E-23 |
| hsa-miR-200a-5p | 0.126667 | 4 | 5.173156 | 1.63E-104 | 6.30E-104 |
| hsa-miR-141-3p | 1.27 | 39.568 | 5.112364 | 0 | 0 |
| hsa-miR-378i | 2.906667 | 81.63 | 4.948729 | 0 | 0 |
| hsa-miR-1245b-3p | 0.001 | 0.12 | 4.379607 | 0.000341 | 0.00037 |
| hsa-miR-200a-3p | 4.663333 | 85.168 | 4.362833 | 0 | 0 |
| hsa-miR-200c-3p | 8.936667 | 150.066 | 4.220714 | 0 | 0 |
| hsa-miR-378e | 22.75 | 368.328 | 4.15812 | 0 | 0 |
| hsa-miR-141-5p | 0.066667 | 0.7882 | 3.823213 | 2.73E-19 | 5.69E-19 |
| hsa-miR-375 | 1.573333 | 20.338 | 3.812566 | 0 | 0 |
| hsa-miR-548az-5p | 0.027 | 0.294 | 3.642641 | 1.57E-07 | 2.22E-07 |
| hsa-miR-5703 | 0.014 | 0.1504 | 3.642641 | 0.000209 | 0.000232 |

**Table S5. Top 20 downregulated miRNA in EAT samples of CAD patients.**

| **miRNA id** | **Expression**  **(CON)** | **Expression**  **(CAD)** | **log2Ratio**  **(CAD/CON)** | **P value** | **Q value** |
| --- | --- | --- | --- | --- | --- |
| hsa-miR-4707-3p | 5.263333 | 0.0306 | -7.26199 | 1.20E-117 | 4.98E-117 |
| hsa-miR-517a-3p | 1.693333 | 0.272 | -2.5691 | 5.28E-26 | 1.27E-25 |
| hsa-miR-7975 | 4.583333 | 1.042 | -2.00706 | 1.17E-47 | 3.47E-47 |
| hsa-miR-377-3p | 14.94333 | 4.104 | -1.73789 | 3.03E-126 | 1.30E-125 |
| hsa-miR-299-5p | 6.036667 | 1.832 | -1.57358 | 2.19E-45 | 6.50E-45 |
| hsa-miR-143-5p | 10.83667 | 3.606 | -1.49389 | 2.14E-74 | 7.20E-74 |
| hsa-miR-144-3p | 1100.903 | 364.964 | -1.48049 | 0 | 0 |
| hsa-miR-6087 | 3.283333 | 1.226 | -1.39998 | 1.23E-22 | 2.76E-22 |
| hsa-miR-324-5p | 56.62 | 20.032 | -1.38276 | 0 | 0 |
| hsa-miR-4492 | 3.373333 | 1.276 | -1.30489 | 5.32E-20 | 1.13E-19 |
| hsa-miR-299-3p | 5.213333 | 1.902 | -1.28897 | 9.16E-30 | 2.32E-29 |
| hsa-miR-423-5p | 97.97 | 36.656 | -1.28863 | 0 | 0 |
| hsa-let-7i-3p | 92.99 | 34.55 | -1.27951 | 0 | 0 |
| hsa-miR-103a-3p | 675.49 | 266.658 | -1.269 | 0 | 0 |
| hsa-miR-144-5p | 23.02333 | 9.47 | -1.17472 | 3.48E-111 | 1.38E-110 |
| hsa-miR-3074-5p | 1725.673 | 721.978 | -1.11473 | 0 | 0 |
| hsa-miR-1260b | 4.75 | 2.046 | -1.11418 | 6.63E-22 | 1.46E-21 |
| hsa-miR-874-3p | 98.12333 | 43.866 | -1.03358 | 0 | 0 |
| hsa-miR-328-3p | 137.7 | 62.178 | -1.02694 | 0 | 0 |
| hsa-miR-1260a | 80.24 | 37.94 | -1.00333 | 1.32E-289 | 7.32E-289 |
