# Supplementary figures and images for: MicroRNA-200b-3p promotes endothelial cell apoptosis by targeting HDAC4 in atherosclerosis
Source: BMC Cardiovasc Disord. 2021 Apr 12;21:172. doi: 10.1186/s12872-021-01980-0 (PMC8042726; doi:10.1186/s12872-021-01980-0)

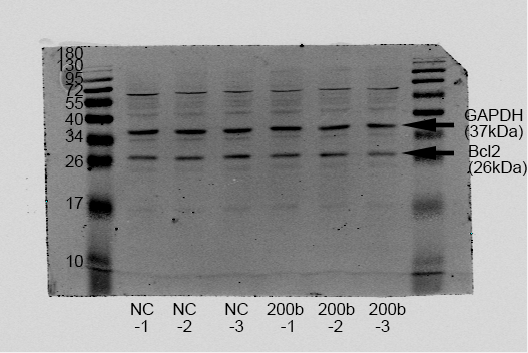

Supplement: Supplementary file 2 — Additional file 2: Figure S1. The full-length blot for figure 4F. [file 12872_2021_1980_MOESM2_ESM.tif]

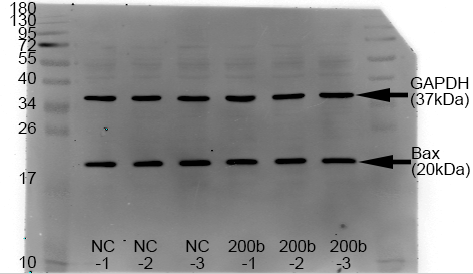

Supplement: Supplementary file 3 — Additional file 3: Figure S2. The full-length blot for Figure 4F. [file 12872_2021_1980_MOESM3_ESM.tif]

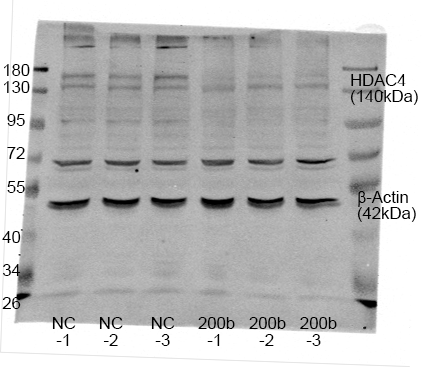

Supplement: Supplementary file 4 — Additional file 4: Figure S3. The full-length blot for Figure 5C. [file 12872_2021_1980_MOESM4_ESM.tif]
